# Pediatric Bacterial Meningitis Surveillance in the World Health Organization African Region Using the Invasive Bacterial Vaccine-Preventable Disease Surveillance Network, 2011–2016

**DOI:** 10.1093/cid/ciz472

**Published:** 2019-08-31

**Authors:** Jason M Mwenda, Elizabeth Soda, Goitom Weldegebriel, Regis Katsande, Joseph Nsiari-Muzeyi Biey, Tieble Traore, Linda de Gouveia, Mignon du Plessis, Anne von Gottberg, Martin Antonio, Brenda Kwambana-Adams, Archibald Worwui, Ryan Gierke, Stephanie Schwartz, Chris van Beneden, Adam Cohen, Fatima Serhan, Fernanda C Lessa

**Affiliations:** 1 World Health Organization Regional Office for Africa, Brazzaville, Republic of Congo; 2 Epidemic Intelligence Service, and, Atlanta, Georgia; 3 Division of Bacterial Diseases, National Center for Immunization and Respiratory Diseases, Centers for Disease Control and Prevention, Atlanta, Georgia; 4 World Health Organization (WHO) Regional Office for Africa, Intercountry Support Team, Harare, Zimbabwe; 5 WHO Regional Office for Africa, Intercountry Support Team, Ouagadougou, Burkina Faso; 6 National Institute for Communicable Diseases, Johannesburg, South Africa; 7 Medical Research Council Unit, The Gambia at London School of Hygiene and Tropical Medicine, Banjul; 8 WHO, Geneva, Switzerland

**Keywords:** pediatric bacterial meningitis, sub-Saharan Africa, case fatality ratios, pneumococcal conjugate vaccine, PCV

## Abstract

**Background:**

Bacterial meningitis is a major cause of morbidity and mortality in sub-Saharan Africa. We analyzed data from the World Health Organization’s (WHO) Invasive Bacterial Vaccine-preventable Diseases Surveillance Network (2011–2016) to describe the epidemiology of laboratory-confirmed *Streptococcus pneumoniae* (Spn), *Neisseria meningitidis*, and *Haemophilus influenzae* meningitis within the WHO African Region. We also evaluated declines in vaccine-type pneumococcal meningitis following pneumococcal conjugate vaccine (PCV) introduction.

**Methods:**

Reports of meningitis in children <5 years old from sentinel surveillance hospitals in 26 countries were classified as suspected, probable, or confirmed. Confirmed meningitis cases were analyzed by age group and subregion (South-East and West-Central). We described case fatality ratios (CFRs), pathogen distribution, and annual changes in serotype and serogroup, including changes in vaccine-type Spn meningitis following PCV introduction.

**Results:**

Among 49 844 reported meningitis cases, 1670 (3.3%) were laboratory-confirmed. Spn (1007/1670 [60.3%]) was the most commonly detected pathogen; vaccine-type Spn meningitis cases declined over time. CFR was the highest for Spn meningitis: 12.9% (46/357) in the South-East subregion and 30.9% (89/288) in the West-Central subregion. Meningitis caused by *N. meningitidis* was more common in West-Central than South-East Africa (321/954 [33.6%] vs 110/716 [15.4%]; *P* < .0001). *Haemophilus influenzae* (232/1670 [13.9%]) was the least prevalent organism.

**Conclusions:**

Spn was the most common cause of pediatric bacterial meningitis in the African region even after reported cases declined following PCV introduction. Sustaining robust surveillance is essential to monitor changes in pathogen distribution and to inform and guide vaccination policies.

## Teaser

We describe the epidemiology of meningitis cases due to *Streptococcus pneumoniae*, *Neisseria meningitidis*, and *Haemophilus influenzae* across 26 African countries participating in the World Health Organization’s Invasive Bacterial Vaccine-preventable Diseases Network, including trends in vaccine-type pneumococcal meningitis post–pneumococcal conjugate vaccine introduction.

Bacterial meningitis is a severe infection associated with high morbidity and mortality in young children, especially in low-income countries [1]. Sub-Saharan Africa has high rates of endemic and epidemic disease, accounting for the world’s greatest burden of meningitis due to *Haemophilus influenzae* type b (Hib), *Neisseria meningitidis* (Nm), and *Streptococcus pneumoniae* (Spn) [2–5].

Over the last decade, countries within the World Health Organization (WHO) African Region introduced vaccines targeting these organisms. Hib conjugate vaccine was first successfully introduced in The Gambia in 1997, followed by a substantial reduction in invasive Hib disease incidence [6]. Through financial support from Gavi, the Vaccine Alliance, all 47 countries within the WHO African Region have introduced Hib-containing vaccine as part of their national childhood Expanded Programme on Immunization (EPI) [7].

A mass vaccination campaign with meningococcal serogroup A conjugate vaccine (MACV, MenAfriVac®) was first implemented in Burkina Faso in 2010, resulting in a 99.8% decline in the risk of serogroup A meningitis [8]. MACV mass vaccination campaigns have since been conducted in 21 countries within the African “meningitis belt,” an area that spans from Ethiopia to Senegal [9]. Seven countries also introduced MACV into their routine childhood immunization schedules beginning in 2016 [4, 10, 11].

Currently, 39 of 47 (83%) countries in the African Region have introduced pneumococcal conjugate vaccine (PCV) into their routine EPI [7]. Post–PCV introduction studies in several African counties have shown significant declines in rates of invasive pneumococcal disease and severe pneumonia [12–16].

Robust disease surveillance systems are essential for guiding further vaccine introduction, monitoring vaccine impact, and describing changes in disease epidemiology over time. In 2001, the African Paediatric Bacterial Meningitis (PBM) surveillance network was initiated with the support of WHO and global immunization partners. This hospital-based sentinel surveillance system collected information on clinically suspected bacterial meningitis cases among children <5 years of age in 31 African countries [17]. In 2008, PBM became part of the global Invasive Bacterial Vaccine-preventable Diseases (IB-VPD) Surveillance Network with other WHO member states [18]. The objectives of the IB-VPD surveillance network include describing the epidemiology and estimating the burden of invasive bacterial vaccine-preventable diseases, establishing a surveillance platform to measure vaccine impact, and characterizing the circulating bacterial serotypes/serogroups among children <5 years old.

We used data collected as part of IB-VPD surveillance in the WHO African Region (2011–2016) to describe laboratory-confirmed meningitis among pediatric patients within the South-East and West-Central African subregions, including pathogen distribution, clinical outcomes, and trends in vaccine-type pneumococcal meningitis following PCV introduction.

## METHODS

Thirty-one of 47 countries in the WHO African Region provided surveillance data for children <60 months old to the IB-VPD network from 2011 through 2016. We excluded neonates (<1 month old) because bacterial etiology of meningitis in this age group is different compared to that of older children and more commonly acquired during birth or hospitalization. Cases were identified as “suspected” based upon the presence of fever and meningeal signs; “probable” based upon cerebrospinal fluid (CSF) appearance, white blood cell count, protein, and glucose; and laboratory-confirmed if *H. influenzae* (Hi), Nm, or Spn was identified by a laboratory test in a child who met the suspected or probable case definition (Supplementary Figure 1). Countries (n = 5) with >50% of records missing data for the classification of suspected meningitis or with <10 suspected meningitis cases across all surveillance years were excluded.

### Data Collection

Trained clinicians at each sentinel surveillance site were responsible for enrolling patients with suspected meningitis. For each patient, a standardized case investigation form containing information on patient demographics, clinical signs/symptoms, outcome, and laboratory results was completed. Data were entered into an Epi Info database and transferred on a monthly basis to Ministries of Health and to WHO Intercountry Support Teams. The Intercountry Support Teams were responsible for data aggregation across countries with subsequent sharing of the data with the WHO Regional Office.

### Laboratory Methods

From most patients with suspected meningitis, a CSF specimen was collected and processed by the sentinel site laboratory for cell count, glucose and protein concentrations, Gram stain, and bacterial culture (when supplies were available). Some laboratories performed rapid diagnostic tests, such as immunochromatographic test for Spn and bacterial latex agglutination testing for Hi, Nm, and Spn.

Most countries within the South-East and West-Central subregions sent CSF specimens to 1 of 2 regional reference laboratories (RRLs): the National Institute for Communicable Diseases (NICD) in South Africa serving the South-East subregion, and the Medical Research Council Unit (MRC) in The Gambia serving the West-Central subregion. Both RRLs perform real-time polymerase chain reaction (PCR) for detection of Hi, Nm, and Spn, with serotyping (Hi, Spn) or serogrouping (Nm) of positive samples using real-time and/or conventional PCR as previously described [19–23]. Different cycle threshold cutoff values were used by each RRL to define positive, negative, and inconclusive results. The MRC used a cycle threshold (Ct) cutoff for pathogen gene detection (Hi = *hpd*; Nm = *ctra* or *sodC*; Spn = *lytA*) of ≤36, whereas NICD used a Ct value ≤35. PCR detection of the human *RNaseP* gene was used by both RRLs to confirm true-negative PCR results, with both laboratories defining negative results as inconclusive with an *RNaseP* Ct value ≥36.

When available, isolates were also sent to the RRLs for confirmation and serotyping/serogrouping by latex agglutination (MRC) or Quellung reaction (NICD).

For patients in whom laboratory results across testing modalities identified >1 pathogen, the PCR result was considered the gold standard and was reported. If >1 pathogen was detected by PCR, contamination was suspected and the result was excluded. For samples not tested by PCR, CSF culture results were prioritized over rapid diagnostic tests.

RRLs submitted all testing results back to the sentinel sites and intercountry support teams. Sentinel sites were responsible for updating RRL sample results into their Epi Info database using unique sample identifiers.

### Statistical Analysis

We analyzed data using SAS version 9.4 software (SAS Institute, Cary, North Carolina). Confirmed meningitis cases were stratified by subregion, age group (1–12 months and 13–59 months), and pathogen. We calculated case fatality ratios (CFRs) for confirmed meningitis cases using data from countries that reported outcome data for ≥5 confirmed cases per age group during 2011–2016.

Annual numbers of Hi and Nm meningitis cases for each subregion, by serotype and serogroup status, respectively, were analyzed for countries that reported data across all surveillance years and had available serogroup/serotype data.

Vaccine-type pneumococcal meningitis was defined as that caused by serotypes included in the 10-valent PCV (PCV10) (1, 4, 5, 6B, 7F, 9V, 14, 18C, 19F, 23F) or the 13-valent PCV (PCV13; including PCV10 serotypes plus 3, 6A, and 19A). Additionally, serotypes that are indistinguishable from PCV10 serotypes by PCR (6A/6B, 7F/7A, 9V/9A, 18C/18F/18B/18A) were classified as PCV10 vaccine serotypes. Annual numbers of overall and vaccine-type Spn meningitis cases were analyzed by subregion for countries that introduced PCV during the surveillance period and met the following criteria: reported Spn serotype data; had data for all surveillance years; had ≥65% PCV coverage for at least 2 consecutive years based upon WHO/United Nations International Children's Emergency Funds estimates; and had at least 2 years of available data following vaccine introduction [24].

We stratified countries meeting the above inclusion criteria by PCV formulation (ie, PCV10 or PCV13). The Cochran-Armitage test for trend was used to evaluate changes in the annual percentage of PCV10 and PCV13 Spn meningitis cases among all Spn meningitis cases with a reported serotype. To confirm observed trends, sensitivity analyses were performed; we excluded data from the United Republic of Tanzania and Ghana because <12 confirmed pneumococcal meningitis cases were serotyped across all surveillance years.

Fisher exact test was used to compare categorical variables. A 2-tailed *P* value of <.05 was considered statistically significant for all statistical tests.

## RESULTS

### Descriptive Epidemiology of Meningitis Cases in the African Region

**Figure 1.**
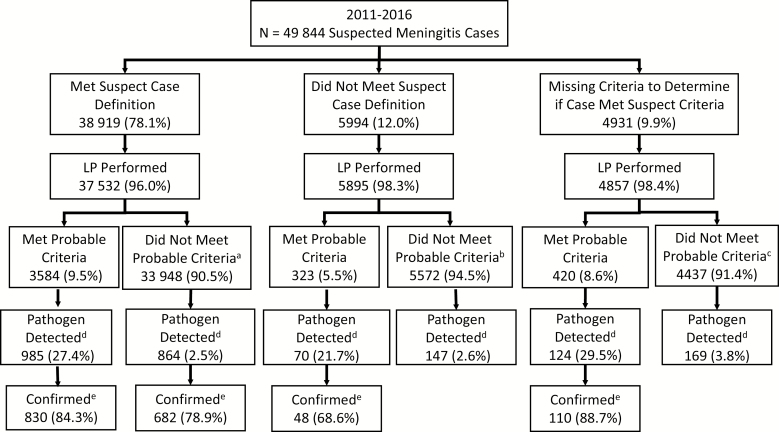
Bacterial meningitis case classification, 2011–2016. Abbreviation: LP, lumbar puncture. Percentage of cases that did not meet probable criteria due to missing data: ^a^1.9%, ^b^1.1%, ^c^1.3%. ^d^*Neisseria meningitidis*, *Streptococcus pneumoniae*, and *Haemophilus influenzae* in addition to other potential pathogens identified by culture or latex agglutination test. ^e^Detection of *N. meningitidis*, *S. pneumoniae*, *and H. influenzae* by polymerase chain reaction, culture, or rapid diagnostic tests (latex agglutination or immunochromatographic test).

We included 26 of the 31 WHO African Region member states participating in the IB-VPD surveillance network during 2011–2016. These encompassed 51 sentinel surveillance sites, including 14 located within the African meningitis belt [4] (Table 1). Of the 49 844 patients with suspected meningitis, 38 919 (78.1%) met the “suspected” case definition, 5994 (12.0%) did not meet this definition, and 4931 (9.9%) were missing data needed to determine suspected status based upon the case definition (Figure 1). A lack of recorded fever was the main reason that patients did not meet the suspected meningitis case definition. Overall, 48 284 (96.9%) patients with suspected meningitis had a lumbar puncture performed. Of those, 1670 (3.5%) had laboratory-confirmed Hi, Nm, or Spn meningitis; 51.4% of the cases were confirmed by PCR and 39.1% by culture (Supplementary Figure 2). Among suspected meningitis cases that did not meet the laboratory-confirmed case definition but had both another pathogen detected and named within the database (n = 155), the most common organisms isolated included *Staphylococcus aureus* (21.9%), *Streptococcus* species (18.7%), *Salmonella* species (12.9%), *Pseudomonas* species (10.3%), and *Klebsiella* species (7.7%).

**Table 1.** Description of World Health Organization African Region Countries Included in the Analysis of the Invasive Bacterial Vaccine-preventable Diseases Surveillance System

Country	Subregion	No. of Surveillance Sites	No. of Surveillance Sites Within the Meningitis Belt [4]	Annual Birth Cohort^a^	Years of Available Data	Year of Hib Introduction	Year of First MACV National Prevention Campaign	Year of PCV Introduction (Formulation)	No. of Suspected Meningitis Cases Reported	No. of CSF Specimens Tested^b^
Ethiopia	SE	3	3	3 256 400	2011–2016	2007	2013	2011 (PCV10)	3810	3808
Lesotho	SE	2	0	61 400	2011–2016	2008	…	2015 (PCV13)	468	303
Madagascar	SE	1	0	825 100	2011–2016	2008	…	2012 (PCV10)	2907	2599
Malawi^c^	SE	1	0	664 000	2011–2012	2002	…	2011 (PCV13)	106	102
Mozambique	SE	3	0	1 123 000	2011, 2013–2016	2009	…	2013 (PCV10)	816	749
Rwanda	SE	2	0	370 700	2011–2016	2002	…	2009 (PCV13)	308	280
Kingdom of Eswatini (formerly Swaziland)	SE	2	0	38 700	2011–2016	2009	…	2014 (PCV13)	432	257
Uganda	SE	3	0	1 748 500	2011–2016	2002	…	2013 (PCV10)	3920	3700
United Republic of Tanzania	SE	3	0	2 122 000	2011–2016	2009	…	2012 (PCV13)	590	515
Zambia	SE	1	0	632 600	2011–2016	2004	…	2013 (PCV10)	2082	1427
Zimbabwe	SE	1	0	535 300	2011–2016	2008	…	2012 (PCV13)	2820	2788
Angola^c^	WC	1	0	1 204 400	2012–2016	2006	…	2013 (PCV13)	1574	1297
Benin	WC	3	3	402 600	2011–2016	2005	2012	2011 (PCV13)	8437	2751
Burkina Faso^c^	WC	1	1	725 700	2011–2016	2006	2010	2013 (PCV13)	1379	1374
Burundi^c^	WC	1	0	444 600	2011–2016	2004	…	2011 (PCV13)	323	35
Cameroon	WC	1	0	850 800	2011–2016	2009	2011	2011 (PCV13)	4263	559
CAR^c^	WC	1	0	164 000	2011–2014, 2016	2008	…	2011 (PCV13)	935	711
Cote d’Ivoire	WC	2	1	872 800	2011, 2013–2016	2009	2014	2014 (PCV13)	1931	1928
DRC	WC	3	0	3 328 900	2011–2016	2009	…	2012 (PCV13)	1373	1109
Gambia^d^	WC	1	1	80 500	2011–2016	1997	2013	2011^c^ (PCV13)	287	255
Ghana	WC	2	0	875 700	2011–2016	2002	2012	2012 (PCV13)	2464	2444
Niger	WC	5	5	995 100	2011–2016	2008	2010	2014 (PCV13)	1844	1363
Nigeria	WC	5	1	7 232 600	2011–2016	2012	2011	2014 (PCV10)	3259	3068
Senegal	WC	1	1	548 600	2011–2016	2005	2012	2013 (PCV13)	639	637
Sierra Leone	WC	1	0	258 800	2011–2014, 2016	2007	…	2011 (PCV13)	259	216
Togo	WC	1	0	258 800	2011–2016	2008	2014	2014 (PCV13)	2618	2550
Total	…	51	14	…	…	…	…	…	49 844	36 825

Abbreviations: CAR, Central African Republic; CSF, cerebrospinal fluid; DRC, Democratic Republic of Congo; Hib, conjugate *Haemophilus influenzae* type B vaccine; MACV, meningococcal serogroup A conjugate vaccine; PCV, pneumococcal conjugate vaccine; PCV10, 10-valent pneumococcal conjugate vaccine; PCV13, 13-valent pneumococcal conjugate vaccine; SE, South-East; WC, West-Central.

^a^World Bank population statistics by country, 2016 (https://data.worldbank.org/indicator/sp.dyn.cbrt.in), Estimates rounded to nearest 100.

^b^Specimens tested by at least 1 laboratory method (polymerase chain reaction [PCR], culture, latex agglutination, immunochromatographic test).

^c^These countries do not send samples to the regional reference laboratories for confirmatory testing by PCR and serotyping.

^d^The Gambia introduced 7-valent PCV in 2009.

Among the 1670 patients with laboratory-confirmed meningitis, 1581 (94.7%) were hospitalized and 189 (11.3%) died.

### Descriptive Epidemiology of Confirmed Meningitis Cases in the South-East and West-Central Subregions

During 2011–2016, 3.9% (716/18 259) of suspected meningitis cases in the South-East subregion and 3.0% (954/31 585) in the West-Central subregion were laboratory-confirmed (Table 2). Spn was the most common pathogen detected in the South-East (499/716 [69.7%]) and West-Central (508/954 [53.2%]) subregions. However, Nm meningitis was more prevalent in the West-Central compared to the South-East subregion (321/954 [33.6%] vs 110/716 [15.4%]; *P* < .0001).

**Table 2.** Confirmed^a^ Pediatric Bacterial Meningitis Cases by Subregion, Age Group, and Pathogen

Subregion and Age Group	No. of CSF Specimens Tested^b^	No. of Confirmed Cases	Confirmed Hi Cases (% of Confirmed Cases)	Confirmed Nm Cases (% of Confirmed Cases)	Confirmed Spn Cases (% of Confirmed Cases)
South-East^c^ (n = 18 259 total suspected meningitis cases)					
1–12 mo	9620	409	63 (15.4)	34 (8.3)	312 (76.2)
13–59 mo	6908	307	44 (14.3)	76 (24.8)	187 (60.9)
Total	16 528	716	107 (14.9)	110 (15.4)	499 (69.7)
West-Central^d^ (n = 31 585 total suspected meningitis cases)					
1–12 mo	8518	495	80 (16.2)	133 (26.9)	282 (57.0)
13–59 mo	11 779	459	45 (9.8)	188 (41.0)	226 (49.2)
Total	20 297	954	125 (13.1)	321 (33.6)	508 (53.2)
Totals of both regions	36 825	1670	232 (13.9)	431 (25.8)	1007 (60.3)

Abbreviations: CSF, cerebrospinal fluid; Hi, *Haemophilus influenzae*; Nm, *Neisseria meningitidis*; Spn, *Streptococcus pneumoniae*.

^a^Confirmed cases refer to suspected or probable bacterial meningitis with laboratory evidence of Hi, Nm, or Spn.

^b^Specimens tested by at least 1 laboratory method (polymerase chain reaction, culture, latex agglutination, immunochromatographic test).

^c^South-East subregion countries: Ethiopia, Lesotho, Madagascar, Malawi, Mozambique, Rwanda, Kingdom of Eswatini (formerly Swaziland), Uganda, United Republic of Tanzania, Zambia, Zimbabwe.

^d^West-Central subregion countries: Angola, Benin, Burkina Faso, Burundi, Cameroon, Central African Republic, Cote d’Ivoire, Democratic Republic of Congo, Gambia, Ghana, Niger, Nigeria, Senegal, Sierra Leone, Togo.

Among laboratory-confirmed cases, outcome data were reported for 68.0% in the South-East and 59.0% in the West-Central subregions. The overall CFR was higher among cases in the West-Central vs the South-East subregion (132/563 [23.4%] vs 54/487 [11.0%]; *P* < .0001) (Table 3). CFR was highest among those infected with Spn in both regions, with higher Spn-specific CFR in the West-Central than the South-East (89/288 [30.9%] vs 46/357 [12.8%]; *P* < .0001) subregion.

**Table 3.** Case Fatality Ratios for Confirmed Cases^a,b^ by Subregion^c^, Age Group, and Pathogen

Subregion and Age Group	Total		Hi		Nm		Spn	
	Deaths	CFR, % (no./No.)	Deaths	CFR, % (no./No.)	Deaths	CFR, % (no./No.)	Deaths	CFR, % (no./No.)
South-East^d^								
1–12 mo	41	14.0 (41/292)	5	11.6 (5/43)	1	5.0 (1/20)	35	15.3 (35/229)
13–59 mo	13	6.7 (13/195)	1	3.3 (1/30)	1	2.7 (1/37)	11	8.6 (11/128)
Total	54	11.1 (54/487)	6	8.2 (6/73)	2	3.5 (2/57)	46	12.9 (46/357)
West-Central^e^								
1–12 mo	59	20.8 (59/284)	7	20.0 (7/35)	9	9.8 (9/92)	43	27.4 (43/157)
13–59 mo	73	26.2 (73/279)	4	12.9 (4/31)	23	19.7 (23/117)	46	35.1 (46/131)
Total	132	23.4 (132/563)	11	16.7 (11/66)	32	15.3 (32/209)	89	30.9 (89/288)
Totals of both regions	186	17.7 (186/1050)	17	12.2 (17/139)	34	12.8 (34/266)	135	20.9 (135/645)

Abbreviations: CFR, case fatality ratio; Hi, *Haemophilus influenzae*; Nm, *Neisseria meningitidis*; Spn, *Streptococcus pneumoniae*.

^a^Confirmed cases refer to suspected or probable bacterial meningitis with laboratory evidence of Spn, Nm, or Hi.

^b^Cases were only included if outcome was known.

^c^Countries that reported <5 confirmed cases with outcome data by age group across all surveillance years were excluded: 1–12 months: United Republic of Tanzania; 13–59 months: Mozambique, Kingdom of Eswatini (formerly Swaziland).

^d^South-East subregion countries: Ethiopia, Lesotho, Madagascar, Malawi, Mozambique, Rwanda, Kingdom of Eswatin (formerly Swaziland), Uganda, United Republic of Tanzania, Zambia, Zimbabwe.

^e^West-Central subregion countries: Angola, Benin, Burkina Faso, Burundi, Cameroon, Central African Republic, Cote d’Ivoire, Democratic Republic of Congo, Gambia, Ghana, Niger, Nigeria, Senegal, Sierra Leone, Togo.

### Annual Numbers of Hi, Nm, and Spn Meningitis Cases by Serotype/Serogroup

**Figure 2.**
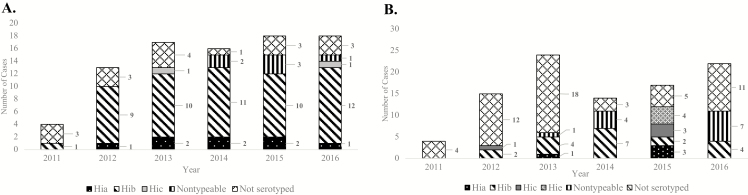
Annual number of confirmed *Haemophilus influenzae* meningitis cases by serotype and African subregion. *A*, Total number of confirmed cases reported by country in South-East Africa: Ethiopia (n = 10), Lesotho (n = 8), Madagascar (n = 5), Kingdom of Eswatini (formerly Swaziland; n = 3), Uganda (n = 36), Zambia (n = 21), and Zimbabwe (n = 3). Confirmed cases from the following countries were excluded: data unavailable for all surveillance years (Malawi, Mozambique); did not report any *H. influenzae* serotype data (Malawi, Rwanda, United Republic of Tanzania). *B*, Number of confirmed cases reported by country in West-Central Africa: Benin (n = 28), Cameroon (n = 15), Gambia (n = 5), Ghana (n = 2), Niger (n = 8), Nigeria (n = 17), Senegal (n = 7), Togo (n = 14). Confirmed cases from the following countries were excluded: data unavailable for all surveillance years (Angola, Central African Republic, Cote d’Ivoire, Sierra Leone); did not report any *H. influenzae* serotype data (Angola, Burkina Faso, Central African Republic, Democratic Republic of Congo). Abbreviations: Hia, *Haemophilus influenzae* type a; Hib, *Haemophilus influenzae* type b; Hic, *Haemophilus influenzae* type c; Hie, *Haemophilus influenzae* type e.

No clear trend in the number of Hi meningitis cases over time was noted in either subregion. Among those with serotype data available, Hib remained the most common serotype; 76.8% (53/69) of Hi cases in the South-East and 44.1% (19/43) of those in the West-Central subregions were Hib (Figure 2A and 2B). At least 1 case of Hib was reported from 14 of the 15 countries included in this analysis.

**Figure 3.**
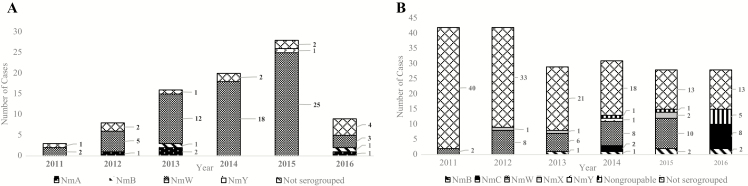
Annual number of confirmed *Neisseria meningitidis* meningitis cases by serogroup and African subregion. *A*, Number of confirmed cases reported by country in South-East Africa: Ethiopia (n = 20), Lesotho (n = 3), Rwanda (n = 5), United Republic of Tanzania (n = 1), Uganda (n = 17), Zambia (n = 37), and Zimbabwe (n = 1). Confirmed cases from the following countries were excluded: data unavailable for all surveillance years (Malawi, Mozambique); did not report any *N. meningitidis* serogroup data (Malawi, Madagascar, Kingdom of Eswatini [formerly Swaziland]). *B*, Number of confirmed cases reported by country in West-Central Africa: Benin (n = 35), Gambia (n = 16), Ghana (n = 12), Niger (n = 82), Nigeria (n = 29), Senegal (n = 26). Confirmed cases from the following countries were excluded: data unavailable for all surveillance years (Angola, Burkina Faso, Central African Republic, Cote d’Ivoire, Sierra Leone); did not report any *N. meningitidis* serogroup data (Angola, Burundi, Central African Republic, Democratic Republic of Congo, Togo). Abbreviations: NmA, *Neisseria meningitidis* serogroup A; NmB, *Neisseria meningitidis* serogroup B; NmW, *Neisseria meningitidis* serogroup W; NmX, *Neisseria meningitidis* serogroup X; NmY, *Neisseria meningitidis* serogroup Y.

Annual number of Nm meningitis cases in the South-East increased through 2015 and then substantially declined in 2016 whereas in the West-Central subregion, case numbers decreased in 2013 and then remained stable (Figure 3A and 3B). Among Nm meningitis cases with serogroup data available, serogroup W was the most common (90.2% [65/72] in the South-East, 54.8% [34/62] in the West-Central subregion). In 2016, serogroup C cases in the West-Central subregion increased. No confirmed cases of serogroup A were identified in the surveillance sentinel sites in the West-Central subregion, although 4 cases were identified in the South-East.

**Figure 4.**
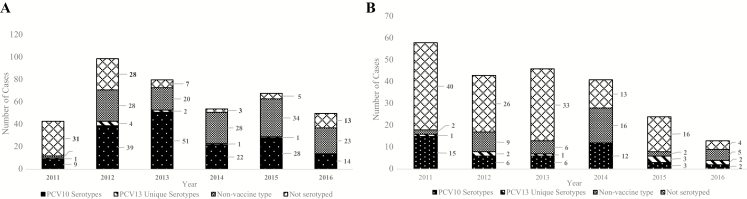
Annual number of confirmed *Streptococcus pneumoniae* meningitis cases by vaccine-associated serotype and African subregion. *A*, Number of confirmed cases reported by country in South-East Africa: Ethiopia (n = 42), Madagascar (n = 81), Kingdom of Eswatini (formerly Swaziland, n = 6), United Republic of Tanzania (n = 6), Uganda (n = 134), Zambia (n = 94), Zimbabwe (n = 31). Confirmed cases from the following countries were excluded: data unavailable for all surveillance years (Malawi, Mozambique); pneumococcal conjugate vaccine (PCV) not introduced during the surveillance period (Rwanda); at least 2 years of data not available following vaccine introduction (Lesotho); did not report any *S. pneumoniae* serotyping data (Malawi). *B*, Number of confirmed cases reported by country in West-Central Africa: Benin (n = 67), Cameroon (n = 57), Ghana (n = 19), Senegal (n = 47), Togo (n = 35). Confirmed cases from the following countries were excluded: data unavailable for all surveillance years (Angola, Central African Republic, Cote d’Ivoire, Sierra Leone); ≤65% PCV coverage for at least 2 consecutive years (Niger, Nigeria); PCV not introduced during the surveillance period (Gambia); did not report any *S. pneumoniae* serotyping data (Angola, Burkina Faso, Burundi, Central African Republic, Democratic Republic of Congo). Thirteen-valent PCV unique serotypes include 3, 6A, and 19A. Abbreviations: PCV10, 10-valent pneumococcal conjugate vaccine; PCV13, 13-valent pneumococcal conjugate vaccine.

Spn meningitis case numbers were highest in 2012 in the South-East and in 2011 in the West-Central followed by an apparent downward trend in both subregions (Figure 4A and 4B). Spn was the most frequently detected pathogen in both subregions over the entire surveillance period. The number of Spn meningitis cases due to the unique PCV13 serotypes (3, 6A, 19A) remained low for both subregions throughout the surveillance period. Among Spn meningitis cases serotyped in the South-East (n = 307) and West-Central (n = 93) subregions, serotype 1 (n = 43 [14.0%]) and serotype 14 (n = 10 [10.8%]) were most prevalent. In the South-East, serotype 1 declined after 2013 whereas serotype 14 decreased after 2011 in the West-Central subregion (data not shown).

**Figure 5.**
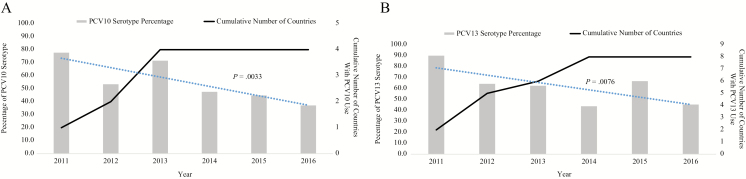
Annual proportion of confirmed *Streptococcus pneumoniae* meningitis cases due to vaccine serotypes by pneumococcal conjugate vaccine (PCV) formulation, 2011–2016. *A*, Proportion of confirmed *S. pneumoniae* meningitis cases due to 10-valent PCV (PCV10) serotypes in countries that introduced PCV10 (n = 4), by year. Of the 6 countries that introduced PCV10, 4 contributed confirmed cases for this analysis: Ethiopia (n = 22), Madagascar (n = 66), Uganda (n = 106), and Zambia (n = 76). Confirmed cases from the following countries were excluded: data unavailable for all surveillance years (Mozambique); ≤65% PCV coverage for at least 2 consecutive years (Nigeria). The percentage of PCV10 serotypes was determined by the number of reported PCV10 serotypes/all reported serotype results in a year: 2011 (7/9); 2012 (31/58); 2013 (48/68); 2014 (20/42); 2015 (26/58); 2016 (13/35). *B*, Proportion of confirmed *S. pneumoniae* meningitis cases due to 13-valent PCV (PCV13) serotypes in countries that introduced PCV13 (n = 8), by year. Of 20 countries that introduced PCV13, 8 were included in this analysis: Benin (n = 20), Cameroon (n = 17), Ghana (n = 8), Senegal (n = 26), Kingdom of Eswatini (formerly Swaziland, n = 3), Togo (n = 22), United Republic of Tanzania (n = 2), and Zimbabwe (n = 21). Confirmed cases from the following countries were excluded: data unavailable for all surveillance years (Angola, Central African Republic, Cote d’Ivoire, Malawi, Sierra Leone); at least 2 years of data not available following vaccine introduction (Lesotho); ≤65% PCV coverage for at least 2 consecutive years (Niger); PCV not introduced during the surveillance period (Gambia, Rwanda); and/or did not report any *S. pneumoniae* serotyping data (Angola, Burkina Faso, Burundi, Central African Republic, Democratic Republic of Congo, Malawi). The percentage of PCV13 serotypes was determined by the number of reported PCV13 serotypes/all reported serotype results in a year: 2011(18/20); 2012 (18/28); 2013 (10/16); 2014 (14/32); 2015 (8/12); 2016 (5/11). Dashed blue line indicates the trend line. Abbreviations: PCV10, 10-valent pneumococcal conjugate vaccine; PCV13, 13-valent pneumococcal conjugate vaccine.

Four countries included in the analysis introduced PCV10 during 2011–2013 and 8 countries introduced PCV13 during 2011–2014 (Figure 5A and 5B). For countries that introduced PCV10, the percentage of vaccine-type meningitis cases decreased from 77.8% (7/9) in 2011 to 37.1% (13/35) in 2016 (*P* = .0033). For countries that introduced PCV13, the percentage of vaccine-type meningitis cases decreased from 90.0% (18/20) in 2011 to 45.5% (5/11) in 2016 (*P* = .0076). The sensitivity analysis revealed similar results.

## DISCUSSION

The IB-VPD surveillance network provides a system for monitoring Hi, Nm, and Spn meningitis infections in children <5 years old across the WHO African Region and highlights the differences and similarities in disease patterns within subregions.

We observed a decline in the proportion of laboratory-confirmed, vaccine-type Spn meningitis in countries that had serotyping data, continuously high PCV coverage, and data available throughout the surveillance period. All of the countries introduced PCV, and all but 2 (Nigeria and Niger) had ≥65% 3-dose vaccine coverage for 2 consecutive years [24]. This decline in vaccine-type Spn meningitis following PCV introduction suggest vaccine effect and are consistent with declines in the burden of pneumococcal disease observed in other countries after PCV introduction [5].

Spn serotypes 1 and 14 were the most prevalent serotypes in the South-East and West-Central subregions, respectively. Both serotypes are associated with invasive pneumococcal disease and are included in PCV10 and PCV13 [25]. In both subregions, the number of cases caused by these serotypes declined over time. Interestingly, serotype 1 was responsible for recent meningitis outbreaks in Ghana despite PCV13 introduction in infants; the highest attack rate was among people ≥5 years old [26, 27]. This could suggest that the vaccine is not providing the desired herd protection (indirect effect) for serotype 1 or that more time is needed to realize the full effects of the vaccination programs. Because the IB-VPD network only collects data among children aged <5 years, we were unable to estimate PCV indirect effects.

Low numbers of Hi meningitis cases were observed over the surveillance period in both subregions, and Hib accounted for the largest proportion of Hi meningitis cases. All but 1 country introduced conjugate Hib vaccine without a booster dose prior to the beginning of our surveillance period. Hib vaccine is effective and the low number of Hi meningitis cases without an increase over time is encouraging. However, continued Hib surveillance is necessary, as vaccine failures with conjugate Hib have been documented and may be more common in countries with a high prevalence of human immunodeficiency virus (HIV) [28].

*Neisseria meningitidis* was more common in the West-Central than in the South-East subregion. There was a notable lack of serogroup A in the West-Central subregion, but a high prevalence of serogroup W and an increase in serogroup C, a serogroup associated with recent outbreaks [29, 30]. The South-East subregion had 4 cases of Nm serogroup A: 3 from Ethiopia (2012, 2013, 2016) confirmed by PCR, and 1 from Lesotho (2013) confirmed by culture. Lesotho has not conducted a MACV campaign. Ethiopia conducted a mass MACV campaign in a phased approach from 2013 to 2015 targeting the age group 1–29 years [31]. The patient vaccination status and province of residence for the NmA cases reported is unknown. Several IB-VPD surveillance sites in the West-Central subregion are in countries within the meningitis belt, areas associated with hyperendemic meningococcal disease with periodic epidemics in the dry season (December–June) [4, 10]. However, since the introduction of MACV there has been a shift from a predominance of serogroup A Nm meningitis to non-A serogroup Nm and Spn meningitis within these areas [26, 30, 32, 33]. There have also been increasing reports of non-A serogroup Nm and Spn outbreaks from areas outside the classic meningitis belt, such as in Liberia and central Ghana [27, 29].

Bacterial meningitis in Africa has been associated with high mortality. These data further demonstrate the high mortality of bacterial meningitis, with CFRs ranging from 12.2% for Hi to 20.9% for Spn. Additionally, we saw that the overall and pathogen-specific CFRs were higher in the West-Central than in the South-East subregion. While our data cannot provide a direct explanation for this finding, greater health disparities may exist for some countries of the West-Central subregion compared to countries in the South-East, which could influence access to healthcare and thus CFR. For instance, the West-Central subregion has demonstrated high maternal mortality, patient dissatisfaction with healthcare facility services, and a need for improved testing and treatment services for people living with HIV—all of which signify challenges within the existing healthcare infrastructure [34–36].

The CFR (12.8%) for Spn in the South-East subregion was lower than previously reported from a PCV introduction study in South Africa (33%) [16, 37]. However, given that countries with a mature PCV vaccination program, such as the United States, have an estimated Spn meningitis CFR of 8%, this value may not be that surprising in the post-PCV era [38]. Our CFR estimates in both subregions are limited by the number of records with reported outcomes. This could result in our CFRs being either an over- or underestimation of the true ratio.

Our data have several other limitations. First, we may have underestimated the number of suspected meningitis cases as we excluded patients without data available to determine whether or not a suspected case definition was met. Second, we excluded 5 countries due to data quality issues. For the remaining countries, missing data limited the determination of suspected or probable bacterial meningitis in some situations. Third, not all samples were sent to the RRLs for PCR testing, which resulted in a large proportion of confirmed cases without available serotyping/serogroup data. Fourth, because this is a sentinel surveillance system involving large tertiary hospitals, the catchment population is difficult to determine and incidence rates could not be calculated. Therefore, we were only able to evaluate the number of cases and proportion of vaccine-type disease over time under the assumption that the catchment populations have remained stable. Fifth, some countries were excluded from trend analyses because they did not report data consistently throughout the surveillance period. Last, by characterizing the PCR indistinguishable Spn serotypes as PCV10 vaccine type, we could be overestimating vaccine serotype and underestimating vaccine impact as this assumes that predominant serotypes within these groups are vaccine-type.

Despite these limitations, the IB-VPD surveillance network is a large surveillance system that can be used to monitor disease in children <5 years of age across the WHO African region. This report is the first comprehensive analysis of IB-VPD meningitis data after widespread Hib, MACV, and PCV introductions in Africa. As the individual countries continue to strengthen data quality and reporting, this system has the potential to not only continue to monitor vaccine impact but to also inform future vaccine policies.

## Supplementary Data

Supplementary materials are available at *Clinical Infectious Diseases* online. Consisting of data provided by the authors to benefit the reader, the posted materials are not copyedited and are the sole responsibility of the authors, so questions or comments should be addressed to the corresponding author.

ciz472_suppl_Supplementary-Figure-1

ciz472_suppl_Supplementary-Figure-2

ciz472_suppl_Supplementary-Figure-Legends

## Notes

***Acknowledgments.*** The authors acknowledge all of the surveillance officers, laboratory personnel, data managers, hospitals, and Ministries of Health that are part of the African Paediatric Bacterial Meningitis surveillance network. The authors also acknowledge the laboratory staff from the regional and global regional reference laboratories the WHO data managers, and to Matthew Westercamp at the Centers for Disease Control and Prevention (CDC) for his help with data management and analyses.

***Financial support.*** Financial support for sentinel site surveillance was provided by Gavi, the Vaccine Alliance, through a grant to the WHO and Ministries of Health of the 31 countries participating in the African Paediatric Bacterial Meningitis Surveillance Network.

***Supplement sponsorship.*** This supplement was supported with funds from Gavi, the Vaccine Alliance through The World Health Organization and the CDC Foundation, and The Medical Research Council Unit The Gambia at the London School of Hygiene and Tropical Medicine.

***Potential conflicts of interest.*** A. v. G. received grants from the WHO Regional Office for Africa during the conduct of the study and has received grants and other fees from Pfizer and Sanofi, outside the submitted work. All other authors report no potential conflicts of interest. All authors have submitted the ICMJE Form for Disclosure of Potential Conflicts of Interest. Conflicts that the editors consider relevant to the content of the manuscript have been disclosed.
