# Supplementary figures and images for: Pediatric Bacterial Meningitis Surveillance in the World Health Organization African Region Using the Invasive Bacterial Vaccine-Preventable Disease Surveillance Network, 2011–2016
Source: Clin Infect Dis. 2019 Aug 31;69(Suppl 2):S49–57. doi: 10.1093/cid/ciz472 (PMC6736400; doi:10.1093/cid/ciz472)

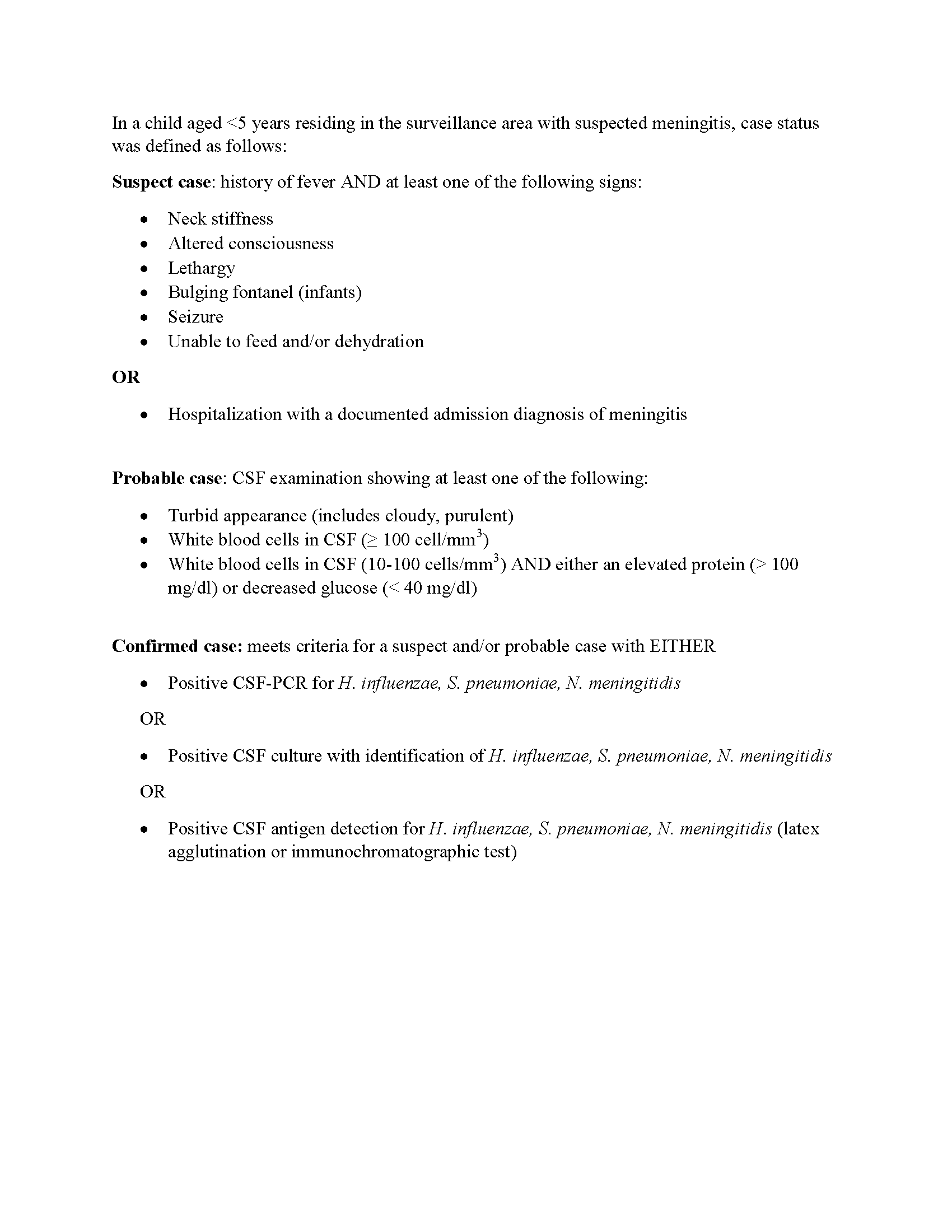

Supplement: ciz472_suppl_Supplementary-Figure-1 [file ciz472_suppl_supplementary-figure-1.png]

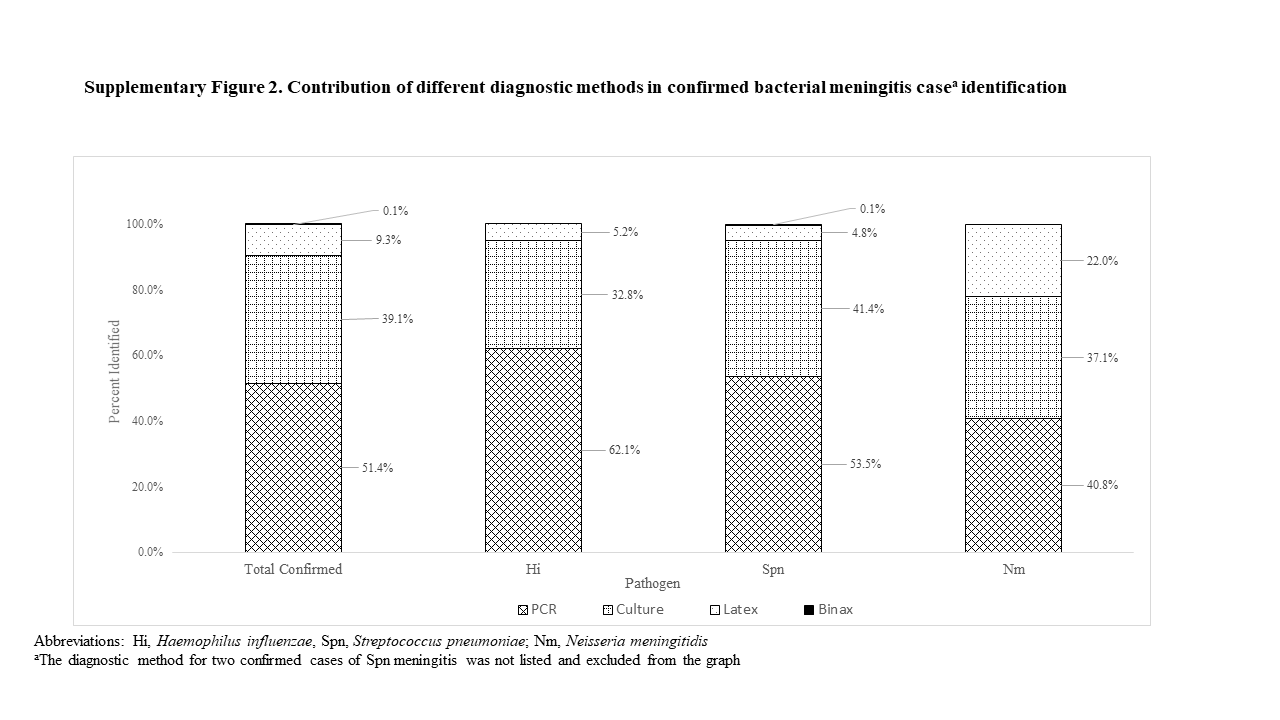

Supplement: ciz472_suppl_Supplementary-Figure-2 [file ciz472_suppl_supplementary-figure-2.png]
