# Supplementary material for: Pediatric Bacterial Meningitis Surveillance in the World Health Organization African Region Using the Invasive Bacterial Vaccine-Preventable Disease Surveillance Network, 2011–2016
Source: Clin Infect Dis. 2019 Aug 31;69(Suppl 2):S49–57. doi: 10.1093/cid/ciz472 (PMC6736400; doi:10.1093/cid/ciz472)
Supplement: ciz472_suppl_Supplementary-Figure-Legends [file ciz472_suppl_supplementary-figure-legends.docx]

**Supplementary Figure Legends**

**Supplementary Figure 1. Bacterial meningitis case definitions**

**Supplementary Figure 2. Contribution of different diagnostic methods in confirmed bacterial meningitis case^a^ identification**

Abbreviations: Hi, *Haemophilus influenzae*, Spn, *Streptococcus pneumoniae*; Nm, *Neisseria meningitidis*

^a^The diagnostic method for two confirmed cases of Spn meningitis was not listed and excluded from the graph
